# Center Frequency Stabilization in Planar Dual-Mode Resonators during Mode-Splitting Control

**DOI:** 10.1038/srep43855

**Published:** 2017-03-08

**Authors:** Adham Naji, Mina H. Soliman

**Affiliations:** 1 Electrical Engineering Department and Centre for Theoretical Physics, The British University in Egypt, El-Sherouk, Cairo, 11837, Egypt; 2 Electrical and Electronic Engineering Department, University of Bristol, Bristol, BS8 1UB, UK; 3 Department of Information Technology and Electrical Engineering, Swiss Federal Institute of Technology (ETH Zurich), Zurich, 8092, Switzerland

## Abstract

Shape symmetry in dual-mode planar electromagnetic resonators results in their ability to host two degenerate resonant modes. As the designer enforces a controllable break in the symmetry, the degeneracy is removed and the two modes couple, exchanging energy and elevating the resonator into its desirable second-order resonance operation. The amount of coupling is controlled by the degree of asymmetry introduced. However, this mode coupling (or splitting) usually comes at a price. The centre frequency of the perturbed resonator is inadvertently drifted from its original value prior to coupling. Maintaining centre frequency stability during mode splitting is a nontrivial geometric design problem. In this paper, we analyse the problem and propose a novel method to compensate for this frequency drift, based on field analysis and perturbation theory, and we validate the solution through a practical design example and measurements. The analytical method used works accurately within the perturbational limit. It may also be used as a starting point for further numerical optimization algorithms, reducing the required computational time during design, when larger perturbations are made to the resonator. In addition to enabling the novel design example presented, it is hoped that the findings will inspire akin designs for other resonator shapes, in different disciplines and applications.

The fundamental tools for a designer to tune the bandwidth or the frequency response shape of a resonant system are based on tuning the coupling coefficients between the resonances that exist in such a system. In dual-mode resonators the operation relies on the coupling between two initially-degenerate modes via a geometric disturbance to the structure’s symmetry^1^,^2^. As the designer controls the severity of the disturbance, the separation between the two resonant mode frequencies varies accordingly. The net effect seen spectrally is a tuning in the separation between the poles (or the equivalent bandwidth) in the resonator’s response, which is sought after during resonator design optimization and in many applications where frequency response tuning is required. However, this utility often comes at a price. On the one hand, the designer’s effected change in the geometry of the structure may lead to a considerable change of the electric impedance (reflection coefficient) seen by the feeding source, depending on the particular geometry in hand and the operating mode, resulting in mismatch losses^3^. On the other hand, the disturbance made in the structure in order to perturb the degeneracy of the modes and convert the resonator into its desired second-order operation is often observed in the frequency domain as a shift more pronounced in one mode than the other. Thus, an increased disturbance may increase the separation between the two resonant frequencies (modes), shifting them in opposite directions, but at different rates, resulting inadvertently in a drift in the center frequency of the dual-mode resonator. Such uneven mode-splitting occurrences are quite commonly seen in practice and in the literature, e.g. refs 4, 5, 6, 7, 8, 9, 10.

Figure 1 shows a common example of this problem. Finding a geometric tuning mechanism that does not have this common phenomenon and that maintains center frequency stability is a nontrivial problem in practice. In fact, in addition to being an interesting geometric design problem in its own right at the resonator level, the ability to maintain a fixed centre frequency may be seen as a welcome feature at the higher system level as it can relatively reduce the effect of impedance mismatch (reflection losses) at the resontor’s terminals during tuning (e.g. one-port network input impedance^11^ is known to be *Z*_in_ = 2*P*_*l*_ + 4*iω(W*_*m*_ − *W*_*e*_)/|*I*|^2^, where *P*_*l*_ is the power lost to the network, *W*_*m*_, *W*_*e*_ are magnetic and electric energies stored in the network’s volume at angular frequency *ω*, and *I* is the current source at the network’s terminals). This would be a desirable practical feature when the resonator is connected to other matching networks or resonators within a larger system.

In this paper we address this geometric design problem and find a novel solution for it. To the authors’ best knowledge, this is a new technique, presented and analysed for the first time in the literature. The paper firstly provides a phenomenological description of the problem, along with a phenomenologically-inspired solution concept that works qualitatively. To further quantify the technique and put it into practice, a more formal treatment is then provided based on perturbation theory. The approximation provided by any perturbation technique is known to work accurately for relatively small disturbances, before it loses validity at larger disturbances. For practical designs that involve larger disturbances, the theory developed herein can be augmented with standard optimization algorithms. The developed theory forms the starting point of such optimization process, shortening its computational time. The field analysis provided helps explaining fundamental phenomena that are not as easily explained using numerical simulations, circuit theory or measurements alone. The theoretical results are found to agree with numerical analysis and practical measurements, confirming the utility of the technique.

It is important to note that the term *planar* here implies no field variations along substrate depth (along *z*, say), which is much smaller a dimension compared to the resonant wavelength. The latter is in the order of the planar surface dimensions (in the *xy* plane) of the resonator. Dominant modes in such structures are assumed to be 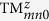 (transverse magnetic) type modes. In other words, a planar resonator is effectively two-dimensional (whereas a transmission line resonator, for example, is effectively one-dimensional). For planar view clarity in all figures, the top conductor of the resonator is shown, but the uniform substrate and ground planes are not. It is also important to note that this study is concerned with the frequency symmetry at the fundamental level of single dual-mode resonator design, not at the higher level of filter design, where *N* such resonators may be coupled together in a system (e.g. with the help of the coupling matrix or other synthesis techniques) and where overall frequency symmetry may be afforded by manipulating coupling configurations at the higher system level. The analysis used in this paper is based on geometric tuning methods, field analysis and perturbation theory (as opposed to electronic device tuning and circuit theory analysis).

## Phenomenological Description and Solution Concept

**Figure 1:**
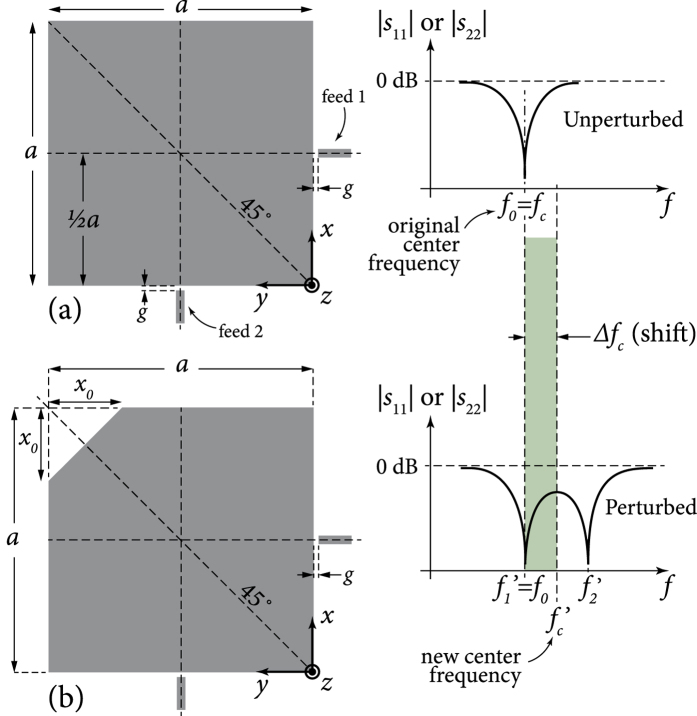
An example of the effect of perturbation on a square dual-mode planar resonator. The center frequency (*f*_*c*_ = *f*_0_) before perturbation (**a**) is shifted up due to the 45°-cut at the corner of the resonator (**b**). This illustrates how the two initially-degenerate modes split into two observable separate modes (rotated by 45 degrees with respect to the original modes) that are not equally shifted around the original center frequency (*f*_0_). The new frequencies after perturbation are *f*′_1_ and *f*′_2_, and the new center frequency is 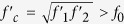. The shown curves are qualitative, to indicate the characteristic behaviour under discussion. Scattering parameters (*S*_*ij*_) are generally used to describe energy reflection (*S*_11_, *S*_22_) and transmission (*S*_12_, *S*_21_) between the ports.

The designer of a planar dual-mode resonator may effect a cut in the body of the resonator or at its outer contour in order to disturb its frequency response and tune the mode-coupling value (the spectral separation between its resonant frequencies, *f*_1_ and *f*_2_, which form dips in the |*S*_11_| response, as in Fig. 1). If the designer wishes to keep the center frequency, *f*_*c*_, of the system fixed during the tuning, the designer will have to devise a mechanism to compensate for the drift incurred in *f*_*c*_ due to the tuning of *f*_1_ and *f*_2_. Most of the time, and for most types of disturbances and cuts, the tuning of *f*_1_ and *f*_2_ will inadvertently affect the center frequency, which is defined by the geometric mean of these two frequencies; 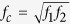. Finding a way to tune these two modes without affecting the center frequency is essentially a geometrical design problem.

To find a solution to this problem, one must first understand the different types of effects that a disturbance can cause in a dual-mode resonator. By analysing these effects and the different phenomena related to them, one can deduce possible mechanisms that can support tuning the mode-coupling while maintaining the center frequency unchanged. For simplicity, and without loss of generality, we base the following discussion and analysis on square dual-mode planar resonators, such as that shown in Fig. 1a, and demonstrate a novel solution example in the microwave regime. The treatment can be extended to other types of structures and frequency regimes, following similar lines of analysis.

### Discussion 

The whole (unperturbed) square resonator has two dominant transverse magnetic degenerate modes (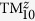 and 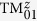) that are spatially orthogonal and uncoupled. They are orthogonal due to the fact that each is aligned to one of the square’s adjacent sides. Each side of the square receives excitation from a corresponding feed (source port). Both modes are centred at the frequency *f*_0_, which is known to be the frequency corresponding to the resonant wavelength *λ*_0_ = 2*a* and the wavenumber *k*_0_ = 2*π/λ*_0_ = *π/a*, where *a* is the side length of the square (Fig. 1a). As an eigenvalue problem, this is described as a problem whose solution is given by a degenerate eigenvalue equal to *k*_0_ with multiplicity of two, and with two corresponding eigenfunctions equal to the field expressions of the first (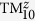) and second (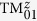) modes:


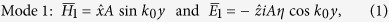



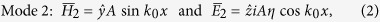


where 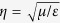 is the wave impedance in the substrate (dielectric) medium of the resonator, *μ* is the permeability, *ε* is the permittivity, *A* is an arbitrary amplitude constant, 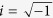, and barred symbols denote vectors.

If a disturbance is now made into this setting, a number of possible phenomena may result, depending on the shape and position of the effected disturbance (or cut). We distinguish here between the phenomena of *mode degeneracy, mode orthogonality* and *mode coupling*, and discuss how they may subtly depend on one another and relate to *mode splitting* and *mode rotation*.

Dual-mode operation necessitates coupling between the two modes, so that they may exchange energy, allowing energy transmission between the two ports and elevating the system to second-order resonance. This requires loss of orthogonality between the two modes, so that one mode has a projection onto the other. Loss of orthogonality, in its turn, requires a disturbance that locally breaks the alignment with the orthogonal axes of the original modes or breaks the symmetry between the two modes (what the modes ‘see’ of perturbations in their respective paths), causing some form of mode rotation. An example of this typically occurs near the edges of the introduced cuts where the boundary conditions need to be satisfied through topical mode rotation.

**Figure 2:**
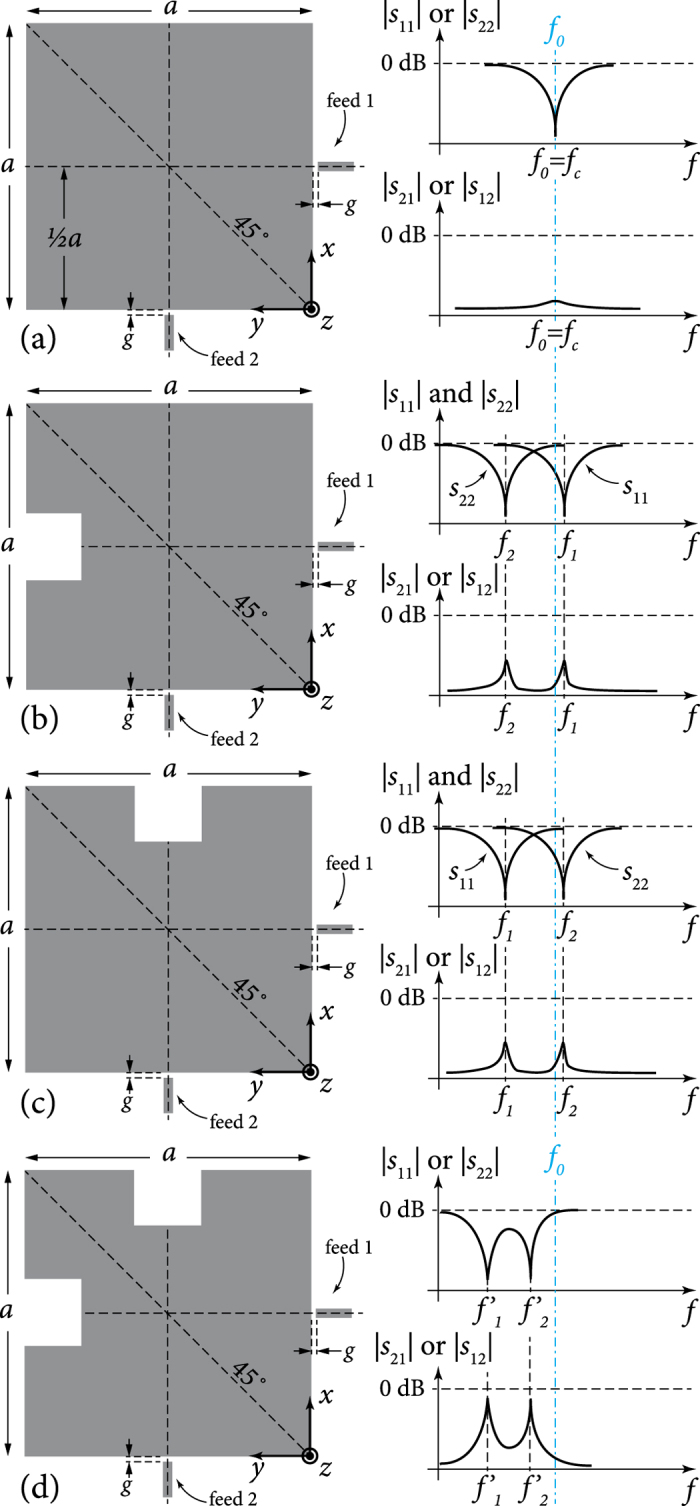
Examples where cuts cause different phenomena in a planar square resonator: (**a**) the unperturbed square with two orthogonal/uncoupled modes resonating simultaneously at *f*_0_; (**b**) a cut that perturbs each mode differently, being an electric cut for mode 1 and a magnetic cut for mode 2, removing mode degeneracy but maintaining overall mode orthogonality with no/low coupling (from local mode rotation) and no mode rotation/splitting; (**c**) similar to case (**b**) but with the cut’s position flipped with respect to the modes (feeds); and (**d**) a combination of cuts (**b**) and (**c**) where both coupling and mode splitting are observed due to the removed symmetry between the modes, which are now rotated by 45 degrees. The first mode’s frequency is denoted *f*_1_ or *f*′_1_, while the second’s is denoted *f*_2_ or *f*′_2_ (primes indicate rotated modes). The shown curves are qualitative, to indicate the characteristic behaviours under discussion.

Mode nondegeneracy, on the other hand, occurs when the two modes resonate at two different frequencies and it might not be sufficient on its own to cause significant mode coupling. Nondegeneracy requires breaking of the symmetry between the two modes, so that they are not experiencing the same geometrical features in their characteristic (eigen) paths. For example, cuts with different relative shapes or alignments in the paths of each mode may give different frequencies (eigenvalues) while maintaining overall mode orthogonality, which prevents the modes from coupling strongly together, as in Fig. 2b,c. Here, only a mild or nonsignificant coupling effect is seen since only limited local field rotation effects take place, near the edges of the cuts that require the 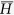 field to be perpendicular to them. However, the overall modal symmetry/orthogonality is not sufficiently broken as to warrant overall (and not just local) rotation of the modes, which would have facilitated greater coupling between them.

**Figure 3:**
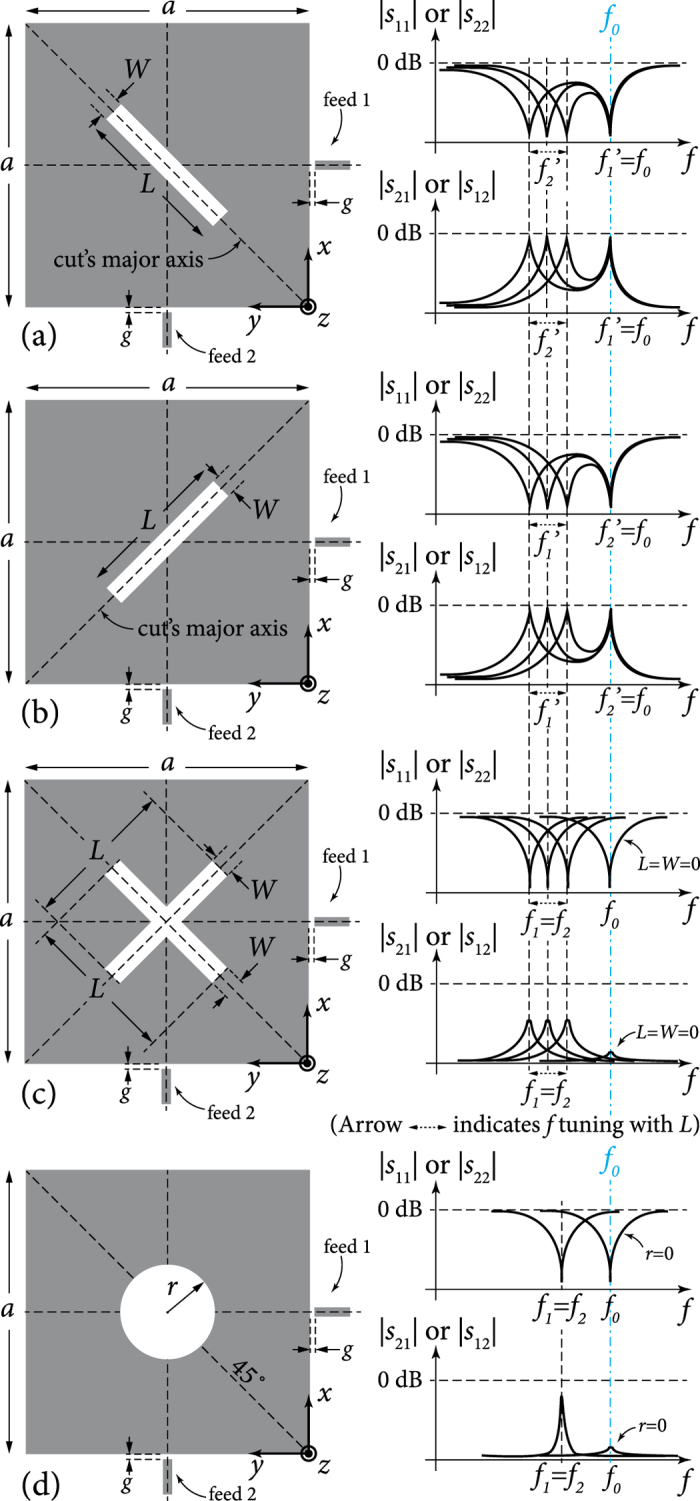
Examples where cuts may cause coupling, but not necessarily mode splitting: (**a**) a symmetry-breaking magnetic perturbation (M-cut) that rotates the modes by 45° and causes coupling, with one rotated mode perturbed significantly while the other largely not, and the net effect being mode splitting; (**b**) a case similar to (**a**) but flipped as to affect a different mode, with same overall effects; (**c**) a cross cut that poses exactly the same kind of M-cuts to each mode, but no breaking of overall symmetry (degeneracy) and no mode splitting, while causing no/low coupling due to the local mode rotation near perturbation edges; and (**d**) a circular cut as another example where some coupling is observed due to local mode rotation at the cut’s boundary, but no mode splitting since symmetry is preserved. The rotated modes’ frequencies are denoted with primed symbols (*f*′_1_ and *f*′_2_). The shown curves are qualitative, to indicate the characteristic behaviours under discussion.

Mode splitting happens when we have both significant mode coupling and nondegeneracy. Mode splitting is seen as a double dipping in each curve for *S*_11_ or *S*_22_. Curves that shift in frequency but without a double dip represent perturbed, but not split, modes. The strength of coupling (due to the local asymmetry or misalignment caused by the perturbation) is seen in the *S*_12_ or *S*_21_ curves. Note that mode nondegeneracy (*f*_1_ ≠ *f*_2_) alone does not necessarily imply mode splitting, since sufficient mode coupling is also required to observe mode splitting. Figures 2 and 3 provide some examples of different scenarios that demonstrate these different phenomena.

The net effect observed from all such phenomena is that mode splitting is synonymous with having both significant mode coupling and mode nondegeneracy.

Since we are interested in proper dual-mode operation with tuning effect in *f*_1_ and *f*_2_, we limit the discussion from now on to structures that can support mode splitting.

### Mode-splitting structure to support center frequency stability

**Figure 4:**
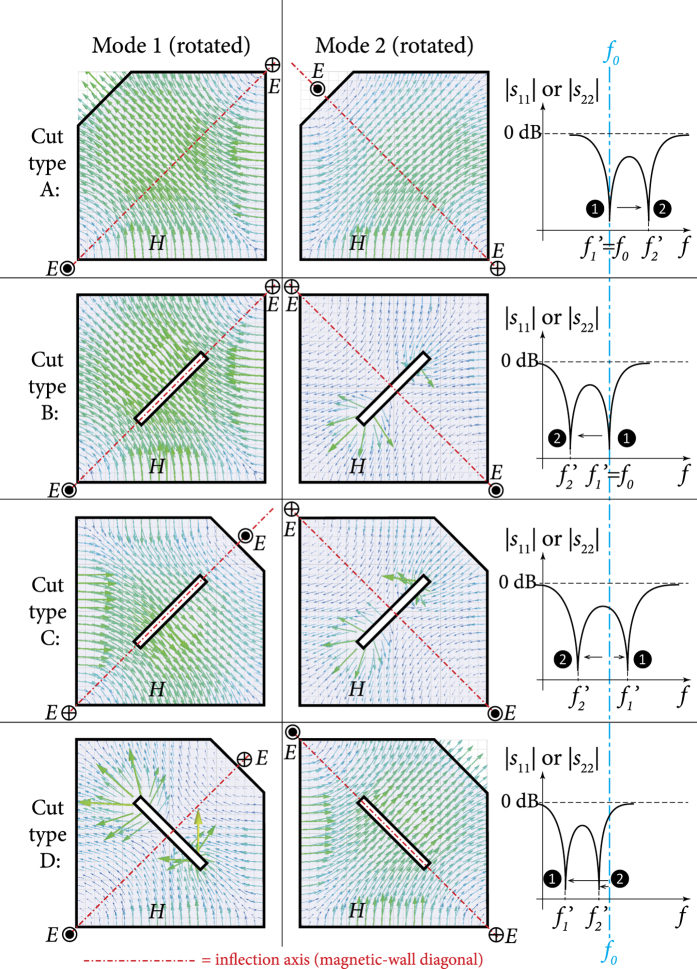
Four cut types that support mode rotation/splitting operation differently: in cut type A, mode 1 is unaffected (due to the its orthogonal alignment relative to the cut’s major axis) while mode 2 is affected by an E-cut that increases its frequency; in cut type B, mode 1 is unaffected while mode 2 is affected by an M-cut that decreases its frequency; in cut type C, mode 1 is affected by an E-cut while mode 2 is affected by an M-cut, causing each one to shift in opposite direction; and in cut type D, mode 1 is affected by a mixed E-/H-cuts while mode 2 is unaffected (except slightly by a small M-cut effect, if cut is large enough) and the frequency shifts are not in opposite directions. Primed symbols (*f*′_1_ and *f*′_2_) indicate rotated modes.

Consider now Fig. 4 of various cut types, all of which support mode splitting. We know from perturbation theory in planar resonators that a cut that causes more loss in the electrical stored energy compared to the magnetic stored energy (call it an E-cut) will result in an increase in resonant frequency, whereas an opposite type of cut (M-cut) will cause a frequency decrease^12^. For each of the four cut types in the figure, the streamlines of the magnetic field of the two rotated modes are shown. We prefer visualizing this problem through the perspective of the magnetic field, since the closed loops of such field may be easier to think of wrapping around the resonator structure and perpendicularly to the resonator’s and cut’s magnetic side-walls. The more rudimentary, but correct, observations of Faraday on field lines tending to shorten and widen themselves whenever possible^13^, may also be helpful when imagined with these 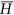 streamlines in the planar substrate. For the most part, the 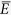 field remains directed with *z* (except for small fringing effects near magnetic walls), whereas 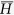 is more obviously rotated in 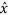 and 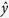 directions when disturbances occur in plane, to meet the boundary conditions.

Note from Fig. 4 that the axes of inflection of the two modes (the magnetic-wall diagonals that the 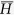 field crosses perpendicularly and about which the fields tend to curl into H-nulls at the far ends where 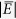 is maximum) are always perpendicular. Only one mode is chiefly perturbed by cut types A and B. In types C and D, cuts A and B are combined to observe the compounded effect on both modes. Only cut type C carries potential for the purpose of center frequency stabilization, since the cuts’ major axes are perpendicular to each other (similar to the modes), causing one mode to be affected by an E-cut (increased frequency above *f*_0_) while the other mode is affected by an M-cut (decreased frequency below *f*_0_). Cut type D fails to achieve a similar behaviour, since the cuts’ major axes are parallel and both affect a single mode in a mixed fashion (E-/M-cuts).

Thus, cut type C is the best candidate in Fig. 4 to support two modes split in different directions, with the potential to maintain a fixed center frequency, if their rates of shifting can be controlled. The larger the cut sizes (*x*_0_ and *L*), the larger the separation between the two frequencies, and vice versa. We now analyse this problem more rigorously, to find the relations between the tuning parameters (i.e. dimensions *x*_0_ and *L*) required to hold the center frequency fixed.

## Technique Formulation

The set of boundaries that exist in this problem makes it belong to the majority of practical cases where finding an exact solution to the partial differential equations governing the fields (Helmholtz’s wave equations) is not possible analytically or in closed form. Particularly, the fact that the boundaries geometry is not compatible with the Cartesian frame of coordinates that describes the contour of the resonator renders the method of separation of variables unusable in this setting. Even if we could assume that the field functions (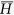 or 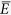) were separable into, say, *g*_1_(*x)g*_2_(*y*), when the 45° cuts are faced in the calculations, aligned along another set of coordinate (*u, v*) that is 45°-rotated with respect to (*x, y*), the conversion of coordinates [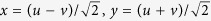] will render the functions *g*_1_(*u, v*) and *g*_2_(*u, v*) inseparable in *u* and *v*. Further, the Helmholtz wave equation is known to be separable only in 11 coordinate systems^14^, most of which belong to confocal coordinates (including the spherical and cylindrical); none of which coincide conveniently with the setup in hand. A possible path to take in such situation is to resort to other analytical methods that solve the problem approximately. Herein we tackle the problem using perturbation techniques. One might argue that modern numerical techniques can solve the problem also approximately. This is true. However, numerical solvers can hardly replace analytical methods in revealing the theoretical concepts behind the operation of a system, assisting our understanding, or indeed finding useful expressions that can be calculated quickly to give a solution to within an acceptable degree of accuracy. In the least, such analytic expressions may be later plugged into a numerical solver as a starting point to optimize the solution further (instead of using numerical brute force only), reducing the overall needed computation time and resources.

We start from the statement of perturbation theory for planar resonators^12^, which gives the relative frequency shift due to a disturbance by


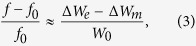


where *f* is the frequency after perturbation, Δ*W*_*m*_ and Δ*W*_*e*_ are the energies removed by the perturbation from the original resonator volume, and *W*_0_ is the total energy (magnetic and electric) stored in the original resonator before any perturbation. Δ*W*_*m*_, Δ*W*_*e*_ and *W*_0_ are given by


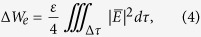



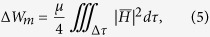



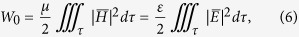


where Δ*τ* is the volume removed by the disturbance from the resonator’s original (unperturbed) volume *τ*. Note that planar resonators may be approximately modelled generally as cavities with magnetic side-walls and electric walls at the top and bottom (where the conductors are)^11^,^12^,^15^. Thus, the tangential component of the magnetic field should vanish at all side walls (parallel to the 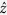 axis), including those caused by cut type C, whereas the electric field must remain parallel to 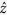 to arrive perpendicular at the top and bottom electric walls. The perturbation effects in these cavities are the dual of those in classical (all-conductor) cavities^12^.

### Discussion on mode expressions

To perform the required calculation in equation (3) we need to find the field expressions that describe as closely as possible the modes that exist in the structure of cut type C in Fig. 4. If we start from the classic Helmholtz equation for the electric field (
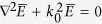
) or magnetic field (
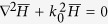
) and apply the unperturbed boundary, we arrive at the dominant modes described by equations (1) and (2). However, such a description is neither the most suitable (for physical understanding) nor the most faithful to the physical reality of having cut features and 45°-rotated modes in the perturbed structure (as seen from Fig. 4). This issue regarding mode representation has been pointed out before in the literature of metallic 3D cavities, where the original nonrotated modes where called ‘nonphysical’ modes because they no longer exist once the structure is perturbed^16^. We therefore choose a set of modes that is rotated by 45° with respect to (1) and (2) by noticing the initial structure’s diagonal symmetry and the alignment of the cuts with the diagonals.

This choice is not only more ‘physical’, but also comes as an expected step from the point of view of functional analysis and perturbation theory for two reasons, one general and one specific to perturbation systems that host mode degeneracy. In general, we know that the natural solutions (eigenfunctions) within the original cavity form a complete orthogonal basis set that spans the solution space of the problem^14^,^17^. As such, these eigenfunctions are linearly independent. This leads to the fact that a general solution for the field propagating inside the cavity, which satisfies the wave Helmholtz equation and boundary conditions, may be reduced to a linear combination of such normalized eigenfunctions (which can be presented by a unitary matrix). The rotated set chosen here is noted to be the simplest such combination, as will be seen from their equations below. Moreover, it is known^18^,^19^,^20^ that a perturbation (*ε*) in a system with mode degeneracy (say two eigenfunctions *ϕ*_1_ and *ϕ*_2_ sharing the same eigenvalue *k*_1_ = *k*_2_ = *k*_0_) will not only break this degeneracy and cause the perturbed modes to be written as linear combination of the original unperturbed cavity modes, but also this effect will hold even as the perturbation is diminished in size. The perturbed eigenfunctions will converge to a linear combination (or rotation) of the unperturbed degenerate modes (*ϕ*′_1_ = *aϕ*_1_ + *bϕ*_2_ and *ϕ*′_2_ = *cϕ*_1_ + *dϕ*_2_) in the limit of very small perturbation (*ε* → 0)^18^. This is a characteristic peculiar to perturbed degenerate modes, which shows that even the smallest perturbation can cause a type of rotation in field representation. For example, in Figs 3a,b and 4, even if we reduce the cut width to very small values (*W* → 0 with arbitrary *L*), the 45°-rotated mode rotation will persist, despite the diminishing slot size.

**Figure 5:**
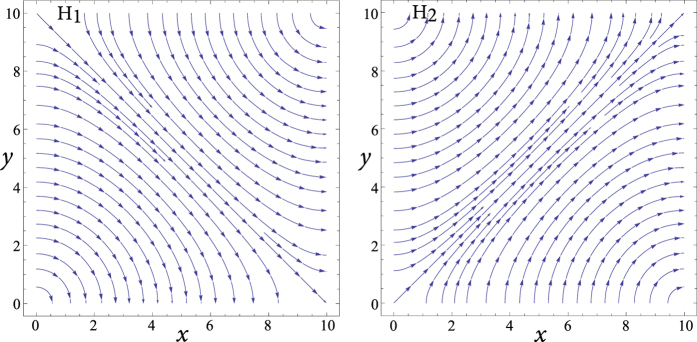
Streamlines of the 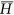 field in the two rotated modes of equations (7) and (9), within a square resonator of arbitrary dimensions, here normalized to 10 × 10.

Since our main interest here is in the dominant modes (first order), for the analysis of cut type C, we choose the following rotated set of modes to perform the calculations:


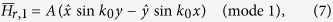



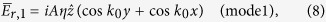



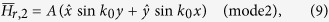



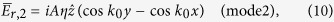


where the subscript ‘*r*’ indicates rotated modes. The 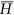 field distribution of these modes is shown in Fig. 5 (the corresponding 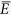 field follows automatically from the magnetic field and Maxwell’s equations).

Note that linear independence and orthogonality are maintained between these two rotated eigenfunctions, similar to the case of the original (unrotated) eigenfunctions. The two sets are thus equivalent and satisfy the wave equation and boundary conditions in the unperturbed structure, but the set along *x, y* becomes nonphysical (would no longer satisfy the wave equation or the overall boundary conditions) after perturbation. The two descriptions can be linked through the relations


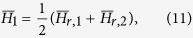



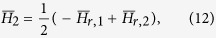



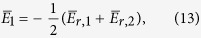



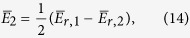


which, in matrix language, is equivalent to basis transformation from vectors aligned along the *x, y* axes to those aligned along the *u, v* axes.

### Solution formulation

**Figure 6:**
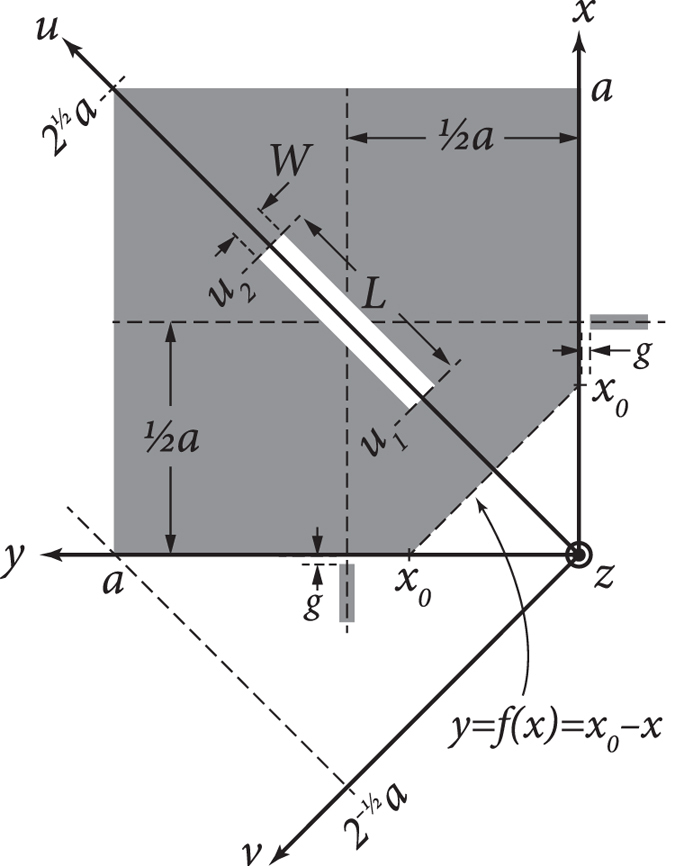
A detailed view of the structure of cut type C.

Now the perturbation of cut type C, which is shown in detail in Fig. 6, is decomposed into two cuts: an inner rectangular cut whose dimensions are *L* × *W* and a corner triangular cut whose sides are equal to *x*_0_. We substitute equations (7),(8),(9),(10) into the integrals (4)–(6) for each of these cuts as the source of the disturbance Δτ, combine the effects of both cuts to find the total energy loss in the 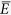 and 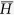 fields for each mode, then substitute the overall result into equation (3) to find the frequency shift for each mode (denoted *f*′_1_ and *f*′_2_ for the rotated modes).

For mode 1 and the inner cut, from equations (4) and (8) and Fig. 6 we have


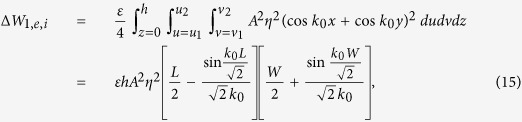



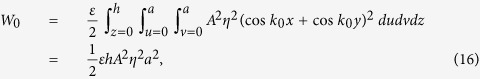


where 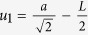, 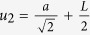, 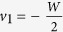, 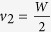, subscript ‘1’ indicates mode 1, and subscript ‘*i*’ indicates the inner cut. In reducing the integrals we used the parametric conversions 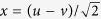 and 
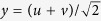
. Note that the result for *W*_0_ is the same for both modes (unperturbed).

Similarly, from equations (5) and (7) and Fig. 6 we have


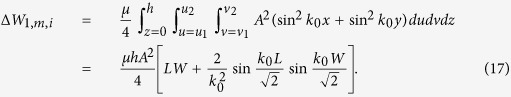


For mode 1 and the corner cut, from equations (4), (5), (7), (8), Fig. 6, and by noticing that the triangular corner cut’s surface integration domain can be expressed as the domain trapped within *x*∈[0, *x*_0_] and *y*∈[0, *f(x*)], where *f(x*) = *x*_0_−*x* is the equation of the slanted side of the triangle (where the metal is severed) as shown in Fig. 6, we have


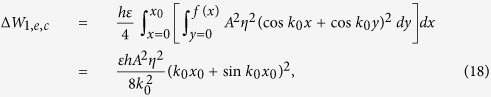



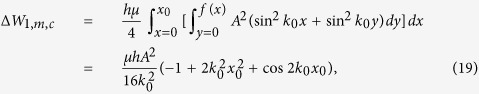


where subscript ‘*c*’ indicates the corner cut.

In a similar fashion, we may find the expressions related to mode 2 to be the following (noting that Δ*W*_2,*m,i*_ = Δ*W*_1,*m,i*_ and Δ*W*_2,*m,c*_ = Δ*W*_1,*m,c*_)


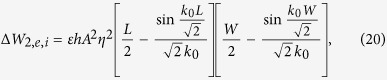



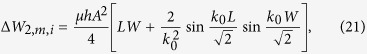



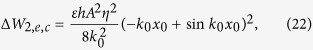



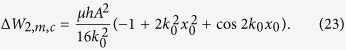


We can now calculate the relative frequency shift due to this perturbation, to within the accuracy of the predictions of perturbation theory, by substituting expressions (15)–(23) into (3). We do this firstly for each individual mode with the inner or the corner cuts, then we combine all effects. The results of substitutions after mathematical manipulation and reduction are


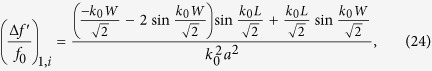



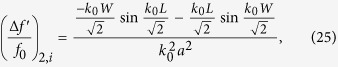



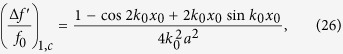



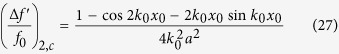


It is seen that combining the frequency effects of the cuts (inner and corner) is merely a simple (linear) sum, since


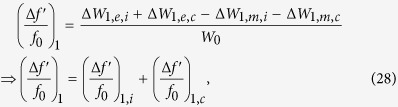



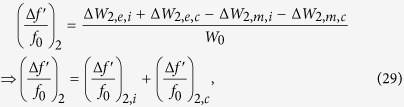


which result in the two equations:


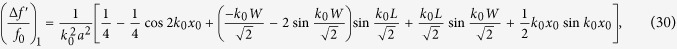



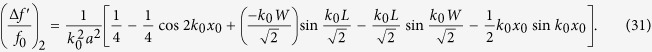


**Figure 7:**
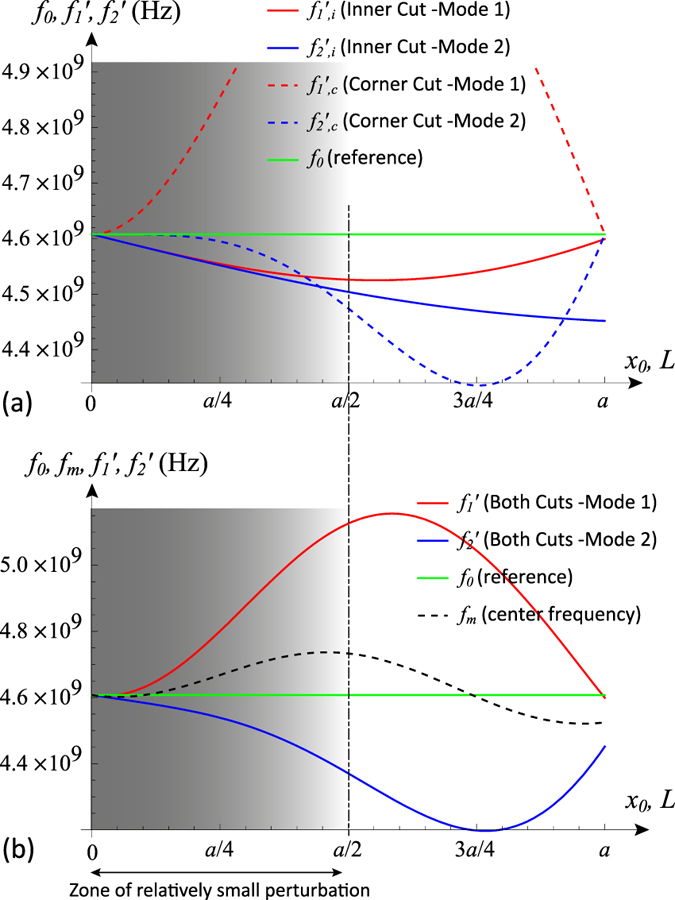
Typical perturbational behaviour of the inner and corner cuts with respect to the modes: (**a**) effect of each cut type on each mode, and (**b**) the combined effect of both cuts on each mode. In this example, the structure has *a* = 20 mm and *W* = 1 mm, with reference (unperturbed) frequency of 4.61 GHz.

Plotting these perturbation relations reveal how the parameters *L, x*_0_ control the frequency shifts (Fig. 7). It is clear from the asymmetric bifurcation of the two modes away from the horizontal line of the reference (unperturbed) frequency *f*_0_ that the the mean frequency will not be fixed at *f*_0_. We note that in systems where the bandwidth is not very wide, the geometric mean 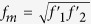 can be effectively approximated by the arithmetic mean *f*_*m*_ ≈ (*f*′_1_ + *f*′_2_)/2, which makes the calculations less cumbersome. Note that *f*′_1_ = *f*_0_ + Δ*f*′_1_ and *f*′_2_ = *f*_0_ + Δ*f*′_2_.

**Figure 8:**
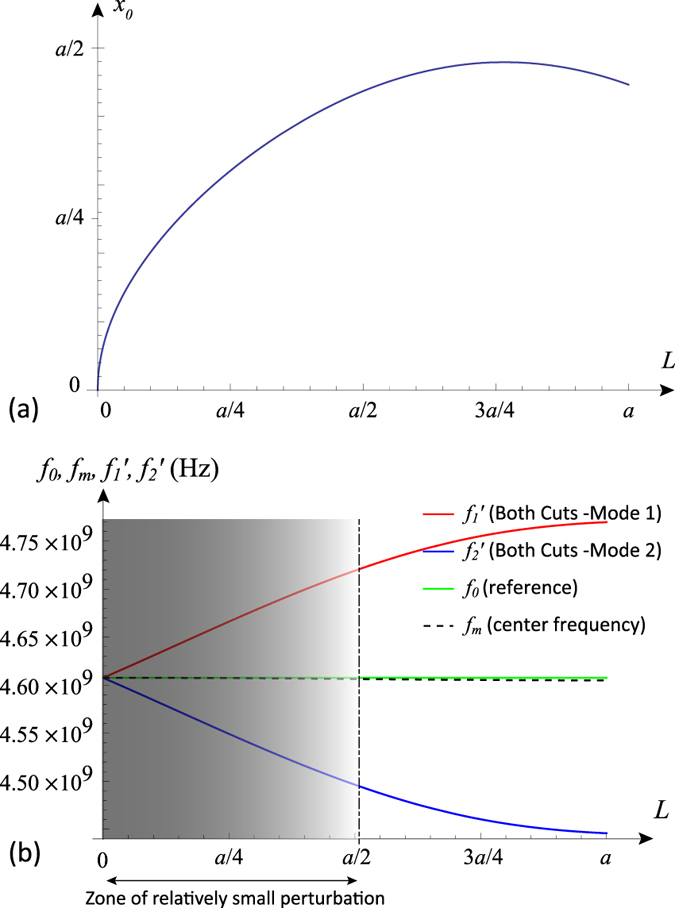
(**a**) Plot of the found perturbational solution (32), which links the parameters *x*_0_ and *L*; and (**b**) the application of the solution, resulting in a stabilized center frequency *f*_*m*_ that matches the reference frequency *f*_0_ (unperturbed). This solution and performance prediction is accurate only within the limit of relatively small perturbation. In this example, the structure has *a* = 20 mm and *W* = 1 mm, with reference (unperturbed) frequency of 4.61 GHz.

We now attempt imposing the center frequency stability condition, by using equations (30) and (31) and insisting that *f*_*m*_ = *f*_0_, which implies that *f*_0_(Δ*f*′_1_ + Δ*f*′_2_) = −Δ*f*′_1_Δ*f*′_2_ or Δ*f*′_1_ ≈ −Δ′*f*_2_. Then, solving (30) and (31) together, we find the sought relation (solution) linking between the parameters *L* and *x*_0_ that will render the bifurcation (splitting) symmetric around the reference and the mean frequency *f*_*m*_ fixed during tuning at *f*_0_:


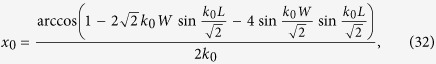


which is plotted in Fig. 8, along with the resulting center frequency stability.

It is important to remember that any perturbation technique is accurate only within the limit of small perturbation effects. In these resonators, this is typically a cut size that is smaller than the resonator dimensions, say <*a*/2 or <*a*/4 in practice, where *a* × *a* is the square size. Since *a* = *λ*_0_/2, this translates into a cut size that is <*λ*_0_/4 or <*λ*_0_/8. Thus, we expect equation (32) to be accurate to within this perturbation validity domain.

We now apply these findings into a practical design and compare measurements with predicted results.

## Implementation Results and Discussion

To implement and validate the found theoretical (perturbational) solution (32), a stripline resonator of cut type C was both numerically analysed and fabricated on a substrate of a relative permittivity of 2 and thickness of 1.52 mm on each side, with square size *a* × *a* = 20 × 20 mm, inner cut width *W* = 1 mm, feedline width 1.2 mm, and a feed coupling gap *g* = 0.1 mm (weak coupling). The inner and corner cuts were then changed in size (*x*_0_, *L*) according to the perturbational solution (32), which links them both in such a way that maintains a fixed center frequency, to within the perturbational limit of accuracy.

**Figure 9:**
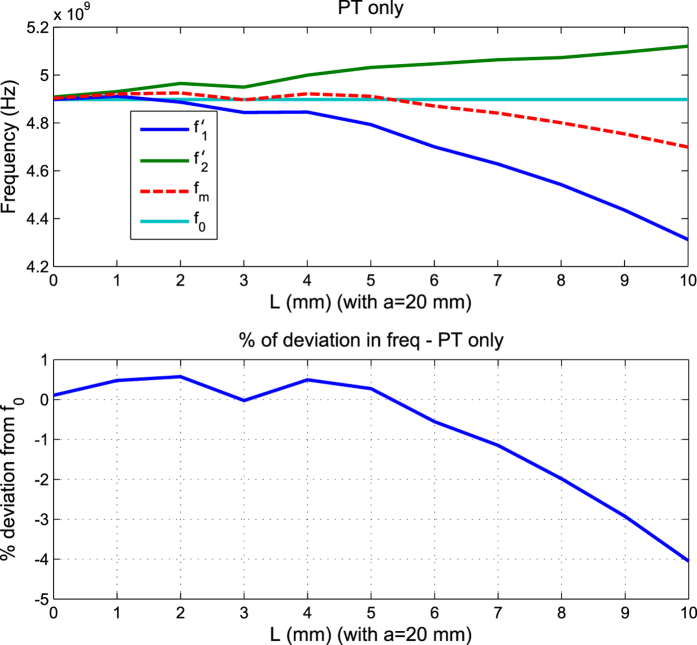
A verification of the solution suggested by Perturbation Theory (PT) in equation (32) using FEM numerical analysis. As expected, the perturbational method is accurate to within the limit of small disturbance. By comparing the center frequency *f*_*m*_ with the reference *f*_0_ (unperturbed), the deviation of the perturbational solution is seen to be within 1% in the domain 0 ≤ *L*<*a*/3, and within 4% in the domain *a*/3 ≤ *L* ≤ *a*/2.

To validate the solution (32) and check the limits of its accuracy, the structure was first solved numerically through the Finite Element Method (FEM), with numerical error less than 1%, using the commercial package of Ansys HFSS. The results are shown in Fig. 9.

It is seen from Fig. 9 that, as expected from the perturbation theory discussed above, the center frequency is fairly stable for most of the tuning range, with deviation within 1% up to about one third of the resonator size (0 ≤ *L* ≤ *a*/3 = *λ*_0_/6) and within 4% deviation as the cut size approach half the size of the resonator (*a*/3 = *λ*_0_/6 < *L* ≤ *a*/2 = *λ*_0_/4).

**Figure 10:**
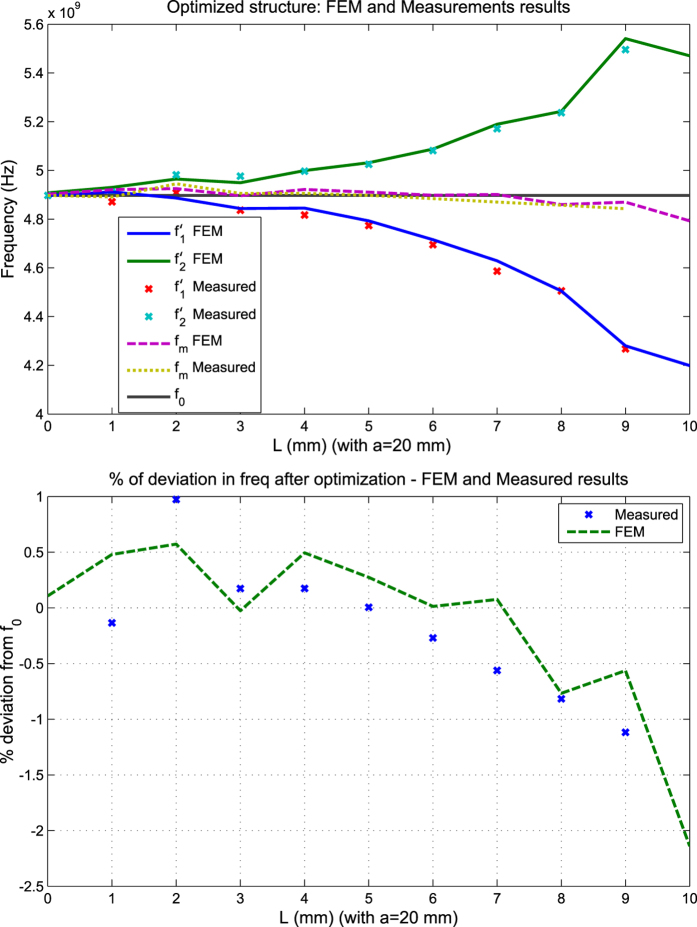
Comparison between numerical FEM predictions and measurements for the structure after using both the perturbational solution (for the domain 0 ≤ *L* ≤ *a*/4) and numerical optimization (for the domain *a*/4<L ≤ *a*/2). The optimization was assisted by the perturbational solution of equation (32) as a starting point. It is seen that the deviation over most of the domain is now within 1% only, with the worst deviation around the *a*/2 end now within 2% (compared to 4% in Fig. 9 when using PT alone).

**Figure 11:**
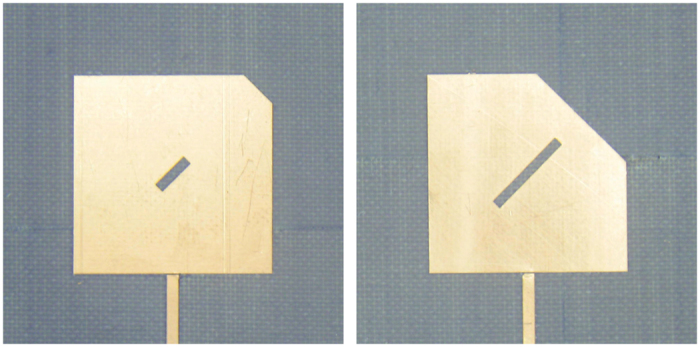
Indicative photographs of two design instances of cut type C.

As an example where optimization could be added to further improve the solution’s accuracy for larger cut sizes, we used a standard quasi-Newton numerical optimization method to improve the error in the range of *L* ∈ [*a*/4, *a*/2], i.e. roughly the half-domain where the original formula started to give deviations higher than 1%. For a linear step of 1 mm in *L* scanning the entire domain between 0 and 10 mm, the corresponding *x*_0_ values were divided into two sets. The first five were taken unchanged from the theoretical result in equation (32), while the second five were found from optimization to be, respectively: 3.21, 4.13, 5.4, 6, 9 and 8.17 mm. To simplify fabrication, the precision of the dimensions was kept to within two decimal points (ten microns) and within overall deviation limit of 2% as target. The resulting performance has improved and the deviation is now within 1% for most of the domain, except near the *a*/2 end, where it becomes around 2%, as shown in Fig. 10 along with measurement results. Measurements are in good agreement with predicted results (tolerances of fabrication and material properties are estimated to be within 2%). This hybrid method, starting from the theoretical finding, is much faster than an open search using optimization without initial information. Figure 11 shows photographs of two implementation instances of cut type C.

It is well known that a planar structure such as the one in hand will appear electrically slightly larger than its physical size, due to the fringing fields near the edges. The enlargement factor is experimentally found from FEM and measurements, based on the whole resonator, to be around 1.083. This is also found to agree with standard models on effective size estimation^15^,^21^. This factor must be included in all theoretical calculations to produce accurate predictions.

**Figure 12:**
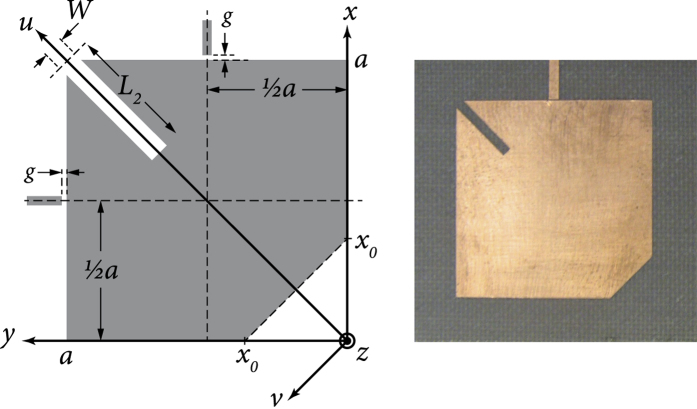
Structure and implementation photograph of an alternative structure that may also be used in practice to stabilize the center frequency during tuning.

Finally, it is important to note that other variants of cut type C may also be used, as long as the the cuts’ relative axes and positions are preserved with respect to the modes and the resonator’s symmetry. For example, another equivalent structure is that shown in Fig. 12, following the same principles discussed above. When the cuts’ sizes (*L* and *x*_0_) become large, this structure may be preferred in practice to avoid early overlapping between the inner and corner cuts.

## Conclusion

We presented a new technique for stabilizing the center frequency of a dual-mode planar resonator during mode-coupling tuning. The technique is analysed using perturbation theory, resulting in a closed-form approximate solution that is accurate within the limit of small perturbation size (here found to be <*λ*_0_/6 for a frequency deviation less than 1%, and <*λ*_0_/4 for a frequency deviation less than 4%). This technique can be useful in many practical applications where tuning of planar dual-mode resonator is required. The found model can be further used to inform numerical optimization techniques when larger perturbations are used in the resonator. Using the model as the optimization’s starting point can reduce the needed computation time and resources. The field analysis provided in this paper may also be useful to inspire similar frequency effects in various structure types and frequency regimes.

## Additional Information

**How to cite this article:** Naji, A. and Soliman, M. H. Center Frequency Stabilization in Planar Dual-Mode Resonators during Mode-Splitting Control. *Sci. Rep.* **7**, 43855; doi: 10.1038/srep43855 (2017).

**Publisher's note:** Springer Nature remains neutral with regard to jurisdictional claims in published maps and institutional affiliations.
